# Lumpy Skin Disease Is Characterized by Severe Multifocal Dermatitis With Necrotizing Fibrinoid Vasculitis Following Experimental Infection

**DOI:** 10.1177/0300985820913268

**Published:** 2020-04-21

**Authors:** Beatriz Sanz-Bernardo, Ismar R. Haga, Najith Wijesiriwardana, Philippa C. Hawes, Jennifer Simpson, Linda R. Morrison, Neil MacIntyre, Emiliana Brocchi, John Atkinson, Andy Haegeman, Kris De Clercq, Karin E. Darpel, Philippa M. Beard

**Affiliations:** 1 The Pirbright Institute, Surrey, UK; 2 The Roslin Institute / Royal (Dick) School of Veterinary Studies, University of Edinburgh, Easter Bush, Midlothian, UK; 3 Istituto Zooprofilattico Sperimentale della Lombardia e dell’Emilia-Romagna (IZSLER), Brescia, Italy; 4 MSD Animal Health, Walton Manor, Walton, Milton Keynes, UK; 5 Sciensano, Exotic and Particular Diseases, Ukkel, Belgium

**Keywords:** bovine, dermatitis, lumpy skin disease virus, poxviridae, skin, transboundary animal diseases, vasculitis

## Abstract

Lumpy skin disease is a high-consequence disease in cattle caused by infection with the poxvirus lumpy skin disease virus (LSDV). The virus is endemic in most countries in Africa and an emerging threat to cattle populations in Europe and Asia. As LSDV spreads into new regions, it is important that signs of disease are recognized promptly by animal caregivers. This study describes the gross, microscopic, and ultrastructural changes that occur over time in cattle experimentally challenged with LSDV. Four calves were inoculated with wildtype LSDV and monitored for 19 to 21 days. At 7 days after inoculation, 2 of the 4 cattle developed multifocal cutaneous nodules characteristic of LSD. Some lesions displayed a targetoid appearance. Histologically, intercellular and intracellular edema was present in the epidermis of some nodules. Occasional intracytoplasmic inclusion bodies were identified in keratinocytes. More severe and consistent changes were present in the dermis, with marked histiocytic inflammation and necrotizing fibrinoid vasculitis of dermal vessels, particularly the deep dermal plexus. Chronic lesions consisted of full-thickness necrosis of the dermis and epidermis. Lesions in other body organs were not a major feature of LSD in this study, highlighting the strong cutaneous tropism of this virus. Immunohistochemistry and electron microscopy identified LSDV-infected histiocytes and fibroblasts in the skin nodules of affected cattle. This study highlights the noteworthy lesions of LSDV and how they develop over time.

Poxviruses are large double-stranded DNA viruses that replicate in the cytoplasm of the cell. They cause disease in a wide range of animals and humans. The most famous poxvirus is the orthopoxvirus variola virus, the causative agent of the human disease smallpox. Poxviruses of veterinary importance include orf virus in sheep and goats, myxoma virus in rabbits, fowlpox virus in poultry, and the 3 capripoxvirus species.

The capripoxvirus genus contains 3 species of poxvirus that cause high-consequence transboundary disease in ruminant livestock. Sheeppox virus and goatpox virus cause severe disease in sheep and goats, while lumpy skin disease virus (LSDV) causes disease in cattle and water buffalo. The 3 species are highly host-specific and of particular concern to rural communities in Africa and Asia where outbreaks of disease contribute to food insecurity.

LSDV has traditionally been found in Africa. During the 2012–2018 Eurasian LSD epidemic, the virus expanded its geographical range into the Middle East and Europe, causing disease in countries including Iran, Iraq, Jordan, Turkey, Russia, Kazakhstan, Greece, Albania, Serbia, and Bulgaria. The morbidity and mortality associated with this epidemic were reported at 9% to 26% and 0.5% to 2%.^2,3,19,20,22^ In comparison, data from LSD outbreaks over 15 years in an endemic area in Uganda reported a lower disease impact with 4.77% morbidity and 0.03% mortality.^17^

The characteristic clinical sign of LSD is distinctive and numerous raised cutaneous lesions from 0.5 to 5 cm in diameter that develop over 3 to 4 days from macules to papules to nodules. The cutaneous lesions are often accompanied by oral, nasal, and ocular discharge; lethargy; anorexia; and in lactating animals, a rapid drop in milk production. Brisket edema and superficial lymphadenopathy are also reported. After 1 to 2 weeks, the skin nodules become necrotic, and the center eventually sloughs.^1,5,8,14,18,21^ The aim of this study was to describe the gross, microscopic, and ultrastructural pathology occurring in cattle experimentally inoculated with LSDV.

## Materials and Methods

### Ethical Statement

This work was conducted under license P2137C5BC from the UK Home Office according to the Animals (Scientific Procedures) Act 1986. The study was approved by the Pirbright Institute Animal Welfare and Ethical Review Board.

### Virus

The LSDV strain used in this study was sourced from the OIE Capripoxvirus Reference Laboratory at Pirbright and originated from the skin of an LSD-affected bovine in eastern Europe in 2016. The virus was grown on MDBK cells (ATCC code CCL-22) in high-glucose Dulbecco’s modified Eagle’s medium (Life Technologies No. 41965) supplemented with 2.5% fetal bovine serum (Antibody Production Services Ltd), and 50 μg/ml penicillin-streptomycin (Life Technologies No. 15140122) at 37°C in a 5% CO_2_ atmosphere. Infectious virus titre (number of plaque-forming units/milliliter, PFU/ml) was determined by plaque assay on MDBK cells.

### Antibodies

The monoclonal antibody targeting LSDV (2C6) was secreted into the supernatant of a hybridoma culture maintained in a CELLine disposable bioreactor (Integra). The 2C6 antibody hybridoma was generated by immunizing a mouse with inactivated, partially purified LSDV (strain Neethling) then following standard protocols to generate hybridomas. Primary screening of hybridoma supernatants was by indirect ELISA against the homologous antigen, and secondary screening was by immunofluorescence on LSDV-infected MDBK cells. Clone 2C6 provided strong specific labeling of LSDV-infected cells.

### Animal Study

Five male castrated Holstein-Friesian calves 133 to 149 days old (weight range = 82–112 kg) were included in the study. The animals were sourced from a commercial high health herd and confirmed as negative for BVDV via PCR prior to study commencement. The animals were housed in 1 room (22m^2^) in a high-containment (SAPO4) animal facility at the Pirbright Institute. Bedding material was provided (https://www.mayofarmsystems.co.uk/mayo-mattress-stable-mat/), light/dark cycle was 12:12 h, temperature was held between 10°C to 24°C, and humidity 40% to 70%. Animals were fed concentrated rations twice daily and given ad lib access to hay and water. Environmental enrichment was provided, including rubber toys and a hollow ball stuffed with hay.

Four of the 5 animals (calves Nos. 2–5) were randomly assigned to the treatment group and the remaining animal (calf No. 1) to the untreated group. The 4 treated animals were each inoculated with 3 ml of a LSDV suspension at a concentration of 1 × 10^6^ PFU/ml; 2 ml (2 × 10^6^ PFU) was inoculated intravenously (IV) into the jugular vein, and 1 ml (1 × 10^6^ PFU) injected intradermally (ID) into 2 sites on each side of the neck (0.25 ml in each site). The untreated animal was not inoculated. Each animal was examined daily for clinical signs, including fever, anorexia, depression, and for gross lesions, including cutaneous nodules and lymphadenopathy.

Skin biopsies were carried out on the 4 inoculated animals at 5, 9, 11, 15, 17, and 19 days postinoculation (DPI). Hair was removed from the biopsy site with electric clippers and cleaned with skin wipes containing 2% chlorhexidine in 70% alcohol (Clinell, GAMA Healthcare); 2.5 ml of lignocaine (Lidocaine Hydrochloride injection 2%, Hameln Pharmaceuticals) was injected subcutaneously, and after 10 minutes, a 0.8 cm punch biopsy was taken using a disposable biopsy punch (Integra Miltex). One half of the biopsy tissue was placed into 10% sterile buffered formalin (Merck) for a minimum of 48 hours. One quarter was placed into 4% paraformaldehyde (Santa Cruz Biotechnology, sc-281692). The remaining quarter was stored at –80°C for future studies. Insects were fed on the skin of the 4 inoculated cattle at up to 7 time points during the study. The results of this procedure are reported separately (manuscript in preparation).

The 5 animals were euthanized at 19 to 21 DPI with an overdose of barbiturate solution (Dolethal 200mg/ml Solution for injection, Vetoquinol). A postmortem examination was carried out and tissue samples collected into 10% sterile buffered formalin for a minimum of 48 hours before processing.

### Histopathology and Immunohistochemistry

Tissues were processed to paraffin wax blocks, sectioned at 4 μm, and stained with hematoxylin and eosin (H&E). Martius scarlet blue trichrome stain method was taken from Carleton et al^7^ and originally described in Lendrum et al.^15^

For immunohistochemistry to label LSDV, paraffin sections 4 µm thick were cut onto Superfrost Ultra Plus microscope slides (ThermoScientific), dried overnight at 40°C, then heated at 60°C for 25 minutes. Sections were hydrated through xylene (3 × 2 minutes), ethanol (3 × 2 minures), and distilled water (2 minutes) before being rinsed in TBST (Lab Vision Tris Buffered Saline and Tween 20, Thermofisher TA-999-TT) 3 × 2 minutes. Sections were then incubated overnight with the primary antibody (LSDV monoclonal antibody 2C6 diluted 1/15 in antibody diluent, Leica code AR9352) or a negative control of antibody diluent alone. Peroxidase-Blocking Solution (Dako REAL code S2023) was added for 10 minutes, the sections rinsed in TBST for 3 × 2 minutes, and a secondary anti-mouse antibody conjugated to horse-radish peroxidase (Dako REAL EnVision, code K4007) added for 40 minutes at room temperature. The sections were again rinsed in TBST 3 × 2 minutes, DAB+ Chromagen diluted in substrate buffer (20 μl in 1000 μl; Dako, code K3468) was added for 10 minutes before a final rinse in TBST 3 × 2 minutes prior to counterstaining with Harris Haematoxylin (20 seconds). Sections were then dehydrated, cleared, and mounted.

For immunohistochemistry to label CD68, paraffin-embedded tissues section 4 µm thick were prepared and hydrated through xylene (3 × 2 minutes), ethanol (3 × 2 minutes), and distilled water (2 minutes). Antigen retrieval was performed using proteinase K (Dako code S3020) (20 minutes) at room temperature, then sections were rinsed in TBST for 3 × 2 minutes. The primary antibody (Dako mouse anti-CD68 clone EBM11, code M0718) was diluted 1/25 in TBST and incubated on the section overnight at 4°C. The remaining steps were as described previously for the LSDV labeling.

### Electron Microscopy

After 18 days of storage in 4% paraformaldehyde at 4°C, biopsy tissues were cut into 0.5-mm^3^ pieces under EM fixative (2% glutaraldehyde, Agar Scientific, in phosphate buffer) using a dissecting microscope in a fume hood. Tissue pieces were left at room temperature overnight in glutaraldehyde before further processing. The next day, following a 90-minute osmium tetroxide wash (Agar Scientific), samples were thoroughly dehydrated in a graded series of ethanols (70% 45 minutes, 90% 15 minutes, 3 × 100% 15 minutes each) at room temperature and the ethanol then replaced with propylene oxide (Agar Scientific). Infiltration started with 60-minute wash in 50% propylene oxide/50% fresh Agar 100 epoxy resin (Agar Scientific) at room temperature with agitation. After a further 60 minutes in pure resin, samples were placed into Beem capsules (Agar Scientific) and polymerized at 60°C overnight. Thin sections (70 nm) were cut using a Leica UC6 ultramicrotome, stained with uranyl acetate and lead citrate in a Leica EM Stain, and imaged at 100 kV in a FEI T12 TEM with Tietz 2 k × 2 k CCD camera.

## Results

### Clinical Findings

Four calves (Nos. 2–5) were inoculated with 3 × 10^6^ PFU of wildtype LSDV via ID and IV routes and housed with an uninoculated sentinel calf (No. 1) for the duration of the experiment. The 4 ID inoculation sites on the necks of calves Nos. 2 through 5 were measured each day. A firm, well-circumscribed, raised cutaneous nodule formed at each inoculation site and developed to approximately 4 cm in diameter on each calf by 7 DPI. After 7 DPI, the inoculation sites on calves Nos. 2 and 4 reduced in size and in some cases became inapparent. In calves Nos. 3 and 5, the inoculation sites increased up to 8 cm diameter (Figs. 1, 2) and developed a well-circumscribed sunken necrotic center, which sloughed off by the third week of the experiment.

**Figure:**
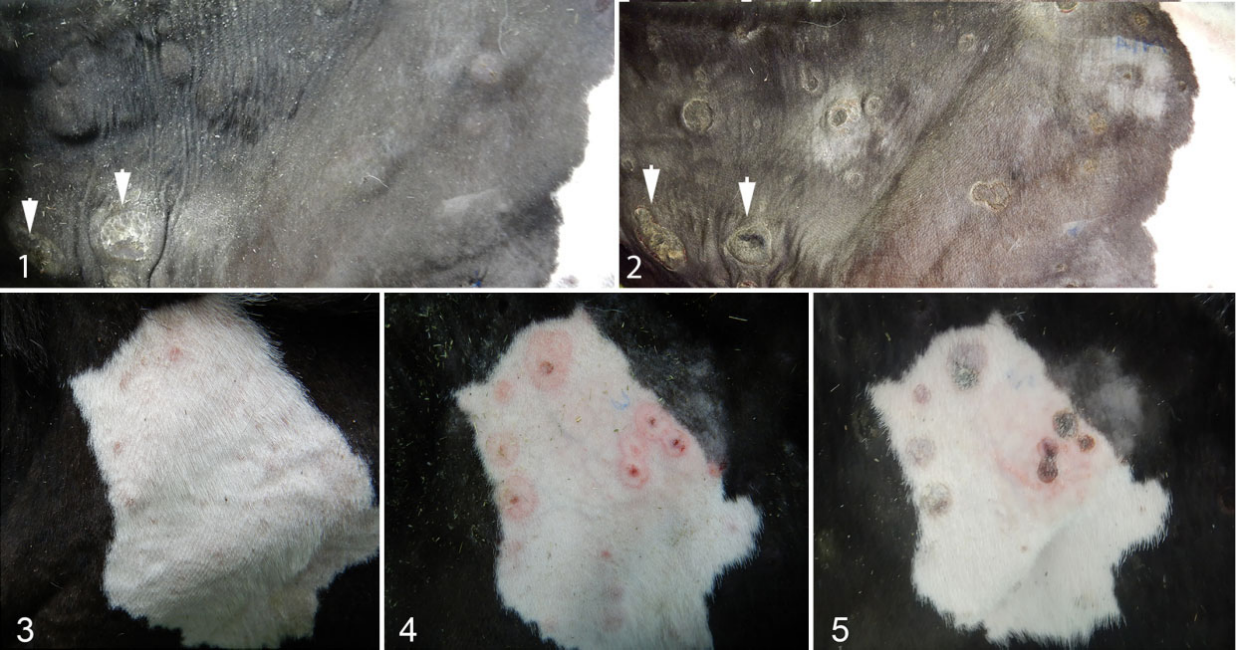
**Figures 1–2.** Lumpy skin disease, calf #5, 12 days post inoculation (DPI) (Fig. 1), and 20 DPI (Fig. 2). Multiple cutaneous nodules are present. Inoculation sites are indicated with arrows. Blue surface labelling (ink) is present on the skin in Figure 2. **Figure 3-5.** Lumpy skin disease, calf #3, right flank. Lesions are present at 7 (figure 3), 13 (figure 4) and 19 (figure 5) DPI. Blue surface labelling (ink) is present on the skin in Figure 4 and 5.

The uninoculated calf No. 1 did not develop any cutaneous lesions during the experiment. Calves Nos. 2 and 4 did not develop any cutaneous lesions apart from the nodules at the sites of intradermal virus inoculation on the neck. In contrast, calves Nos. 3 and 5 developed numerous cutaneous nodules all over the body, particularly on the rostrum, neck, dorsal and lateral body, and legs. Nodules first appeared at 7 DPI (calf No. 3) and 6 DPI (calf No. 5) and continued to appear throughout the study until euthanasia at 19 DPI (calf No. 3) or 20 DPI (calf No. 5). The skin nodules were initially slightly raised, firm, faintly red areas that developed into raised, firm, well-circumscribed nodules up to 3 cm diameter, which, over 1 to 2 weeks, formed a dried, dark red to black sunken central necrotic region that began to peel off (Figs. 1, 2). A minority of the cutaneous lesions displayed a targetoid appearance, with a red center less than 0.5 cm diameter surrounded by a zone of lighter pink discoloration up to 1 cm in diameter, surrounded by a well-demarcated dark red line (Figs. 3–5). The targetoid lesions remained flat and eventually became discolored dark brown to black, consistent with necrosis.

All 4 inoculated calves exhibited moderately to markedly enlarged prescapular lymph nodes that became apparent 5 DPI (calves Nos. 2 and 3) and 4 DPI (calves Nos. 4 and 5). Generalized lymphadenopathy was not a feature of the disease in this study.

Between 7 and 9 DPI, all 4 inoculated calves exhibited a spike in temperature, rising from a baseline of approximately 39°C to a maximum of 40.6°C. Calf No. 1 (uninoculated control) did not display this temperature spike. The temperature of the nonclinical animals (calves Nos. 2 and 4) returned to baseline by 10 DPI; however, the temperature of the clinically affected calves (calves Nos. 3 and 5) remained high for the remainder of the study. In addition to the cutaneous nodules and pyrexia, the clinically affected calves Nos. 3 and 5 exhibited intermittent mild to moderate depression and mild anorexia from 7 DPI to euthanasia, necessitating treatment with nonsteroidal anti-inflammatory drugs. Calf No. 3 was treated on days 9, 12 to 14, and 18. Calf No. 5 was treated on days 11 to 13 and 19. Treatment with nonsteroidal anti-inflammatory drugs did not consistently result in a reduction in temperature in the calves but did improve the general depression shown by both calves. The body condition score of both calves Nos. 3 and 5 dropped from 3 to 5 to 2 to 5 during the experiment. Both animals exhibited mild intermittent nasal discharge and mildly increased salivation. No other clinical signs were noted.

### Gross Lesions

The 5 calves were euthanized at 19 to 21 DPI, and a postmortem examination carried out. Calves Nos. 3 and 5 both had multifocal, numerous (>50), well-circumscribed, flat to slightly raised, round, firm, red to purple to black, occasionally coalescing, cutaneous nodules up to 3 cm diameter. The nodules were particularly numerous on the neck, dorsal and lateral body, and legs of both animals. Nodules were occasionally present in less obvious areas on calf No. 5, for example the skin adjacent to the anus and within the left external ear canal.

Calf No. 5 had no nodules present in any other body system. Calf No. 3 had 1 nodule (1 cm^3^) present within slightly edematous subcutaneous tissues in the region of the brisket. When the musculature underlying the edematous brisket region was incised, an additional small (<2 cm^3^), focal, red, and slightly firm region was found within the muscle tissue.

All 5 calves had mild, multifocal crusting lesions on the head and neck consistent with dermatophytosis (ringworm), which had been present and unchanged since the animals arrived, and varying degrees (mild to moderate) of cranioventral bronchopneumonia. Both were considered incidental findings.

### Histopathology

The 0.8-cm-diameter round skin biopsies (“punch” biopsies) were taken under local anesthesia from calves Nos. 2 through 5 on 5, 9, 11, 15, 17, 19, and 21 DPI, or until euthanasia. The biopsies sampled cutaneous nodules on calves Nos. 3 and 5 and the corresponding area of normal skin on calves Nos. 2 and 4. The biopsies taken from calves Nos. 2 and 4 displayed no significant findings. The biopsies taken from the unaffected skin of calves Nos. 3 and 5 at 5 DPI were histologically normal. Biopsies taken from skin nodules of calves Nos. 3 and 5 from 9 DPI onward showed lesions consistent with poxvirus infection. In the epidermis of some sections, there was ballooning degeneration indicative of intracellular edema. Keratinocytes had abundant clear to pale eosinophilic cytoplasm and shrunken hyperbasophilic nuclei, and there was microvesicle formation. Spongiosis was also present, including widening of the intercellular spaces and rupture of intercellular bridges (Fig. 6). In addition, necrotic keratinocytes were present within the epidermis, scattered either singly or in small clusters, and were characterized by shrunken appearance and hypereosinophilic cytoplasm and hyperbasophilic nucleus. A minority of the degenerate and necrotic keratinocytes contained a round to spherical, pale to intensely eosinophilic, well-defined intracytoplasmic inclusion body (Figs. 7, 8). Similar changes of epithelial cell degeneration and necrosis, intracellular and intercellular edema, and intracytoplasmic inclusion bodies were occasionally noted in the epithelium lining hair follicles.

**Figure:**
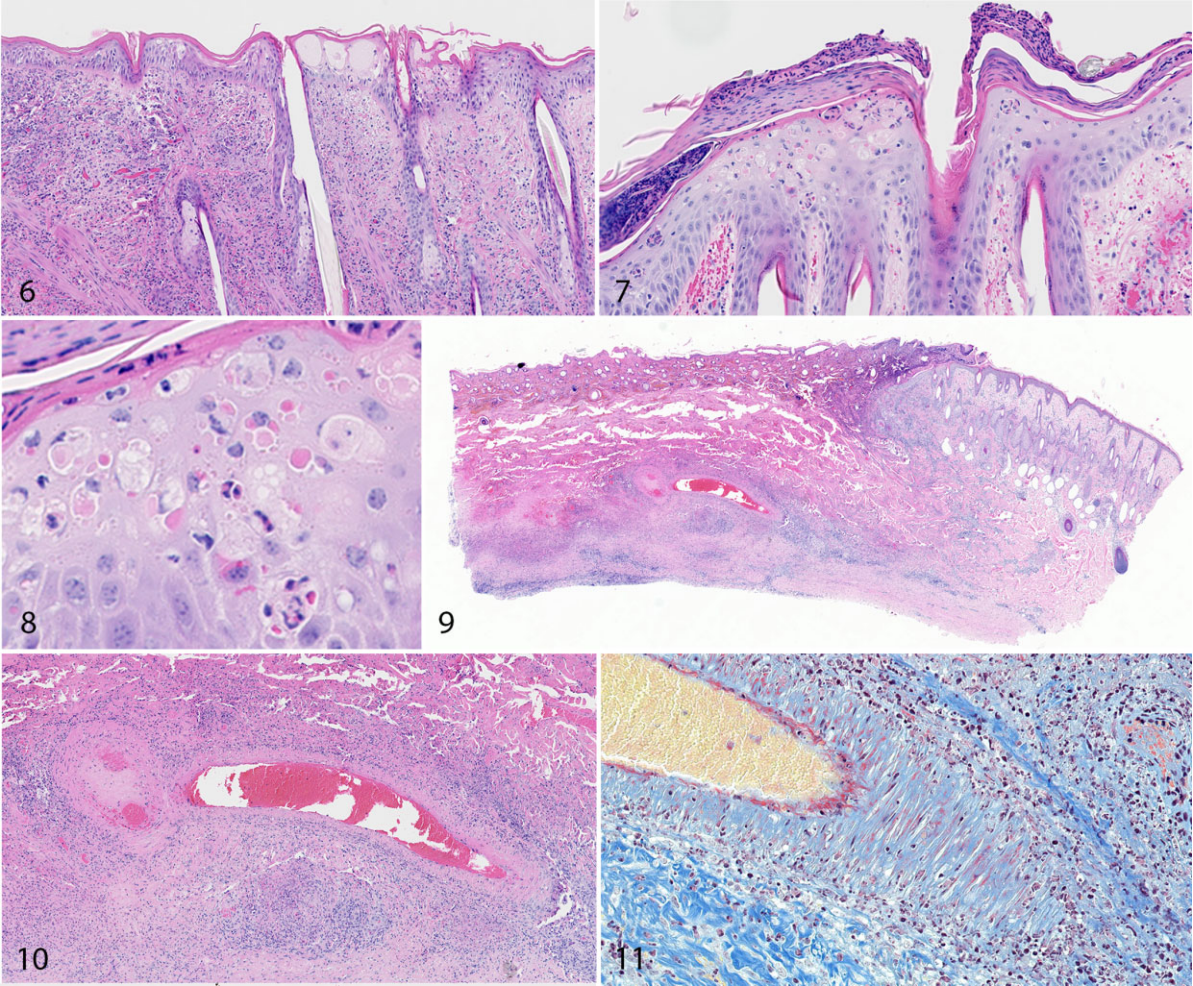
**Figures 6–8.** Acute lumpy skin disease, skin, calf, 9 days post inoculation (DPI; Fig. 6) and 15 DPI (Figures 7, 8). Degeneration and necrosis of keratinocytes, intracytoplasmic inclusion bodies, and vesicles are present in the epidermis. Edema, hemorrhage, and influx of lymphocytes and macrophages are present in the dermis. HE. **Figures 9–11.** Lumpy skin disease, skin, calf. There is a well-demarcated wedge-shaped region of necrosis (infarct; Fig. 9). In the center of the infarct, the wall of a large muscular blood vessel is disrupted by mononuclear inflammatory cells and fibrin (fibrinonecrotic vasculitis; Figure 10 and red staining in Figure 11). HE (Figs. 9, 10). Martius scarlet blue trichrome stain (Fig. 11).

The dermis of early nodular lesions from calves Nos. 3 and 5 was infiltrated with large numbers of large round to elongated histiocytic cells with a large oval, lightly basophilic nucleus and abundant cytoplasm. The histiocytic cells were accompanied by fewer lymphocytes and plasma cells with occasional mild to moderate edema and hemorrhage.

There was marked perivascular accumulation of large histiocytic cells and fewer lymphocytes and plasma cells throughout dermis and particularly focused on the deep cutaneous plexus at the dermal/hypodermal junction. In some sections, the inflammatory cells trafficked into the blood vessel wall and were associated with accumulation of fibrin, as identified by Martius scarlet blue trichrome stain (vasculitis; Figs. 9–11).

Necrosis was a prominent feature of subacute and chronic cutaneous nodules. In well-developed lesions, the dermis and epidermis were entirely replaced by a large well-circumscribed, wedge-shaped block of necrotic tissue exhibiting widespread loss of cellular detail (necrotic sequestra). Large numbers of histiocytic cells and fibroblasts were present at the periphery of the wedge of necrotic tissue.

Samples of skin taken at postmortem examination showed similar changes to the antemortem biopsies, with a spectrum of lesions from abundant histiocytic inflammation in the dermis and deep cutaneous plexus to regionally extensive wedge-shaped areas of epidermal and dermal necrosis (necrotic sequestra; Fig. 12).

**Figure:**
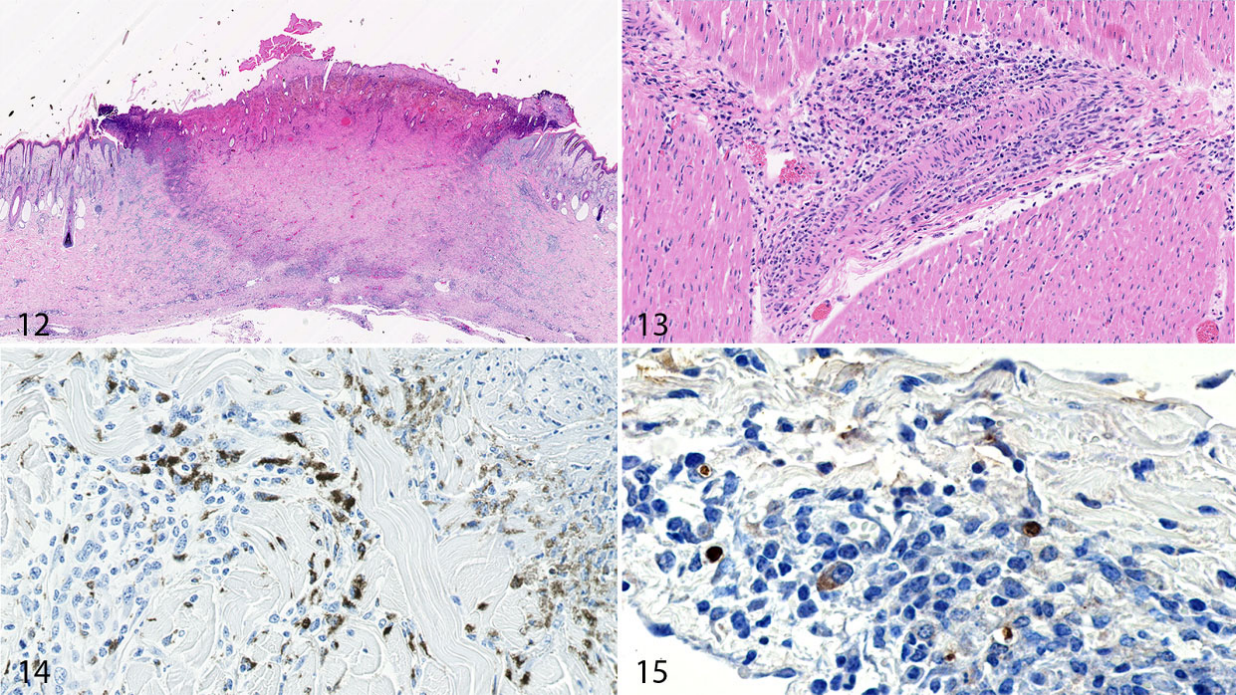
**Figures 12–15.** Lumpy skin disease (LSDV), calf. **Figure 12.** Skin. A cutaneous infarct is present, encompassing both the epidermis and dermis. HE. **Figure 13.** Myocardium. A medium-sized blood vessel in the myocardium is surrounded by histiocytic cells and lymphocytes, with some of these mononuclear cells invading the blood vessel wall. HE. **Figures 14–15.** LSDV, skin, calf. The dermis contains histiocytic cells that are immunolabeled for CD68 (Fig. 14) and for LSDV (Fig. 15).

There were subtle changes identified in other body systems. In calf No. 3, a nodule within the musculature of the brisket region revealed changes consistent with LSDV infection, including vasculitis, necrosis, and intracytoplasmic inclusion bodies within large histiocytic cells and myocytes. In calf No. 5, lesions of small- to medium-sized blood vessels were identified in the kidney, cardiac muscle (Fig. 13), and small intestine. These changes were characterized by perivascular accumulation of large histiocytic cells and fewer lymphocytes. In some cases, the inflammatory cells invaded the wall of the blood vessels and were occasionally accompanied by fibrin accumulation. Only a small number of blood vessels (<4) in each section were affected.

Sections of tissue from the prescapular lymph node (which drained the inoculation site on the neck) contained medullary cords markedly expanded with large histiocytic cells. Germinal centers were prominent and contained tightly packed lymphocytes. There were a small number of necrotic areas.

In summary, histological study of tissues from calves experimentally infected with LSDV revealed a spectrum of skin lesions with changes in the epidermis and dermis and, less commonly, other tissues.

### Immunohistochemistry

An antibody targeting the CD68 molecule on monocyte lineage cells was used to characterize the abundant histiocytic cells identified in the LSDV lesions. Approximately one third of the histiocytic cells in the dermis labeled strongly with the antibody to CD68+ (Fig. 14). A novel anti-LSDV mAb was used to identify cells infected with the virus. Cytoplasmic labeling was identified in a minority of macrophages and epithelial cells, with the labeling often strongest in cytoplasmic vacuoles (Fig. 15). Sections of skin from calf No. 1 (not inoculated with LSDV) and a bovine with cutaneous vasculitis not associated with LSDV were used as negative controls. Neither labeled with the anti-LSDV antibody (data not shown).

### Electron Microscopy

To examine the ultrastructural lesions, affected skin from calf No. 3 at 11 DPI was processed for examination by TEM. Poxvirus virions were recognized by their characteristic hourglass-shaped core surrounded by lateral bodies and enclosed in viral membranes (Figs. 16–19). Virions were present in large histiocytic-like cells and smaller elongated cells (likely fibroblasts). A full spectrum of viral morphogenesis was identified within the infected cells, including spherical immature forms within viral factories, intracellular mature virions with an hourglass core, brick-like intracellular enveloped virions, and extracellular enveloped virions. No virions were identified within endothelial cells.

**Figure:**
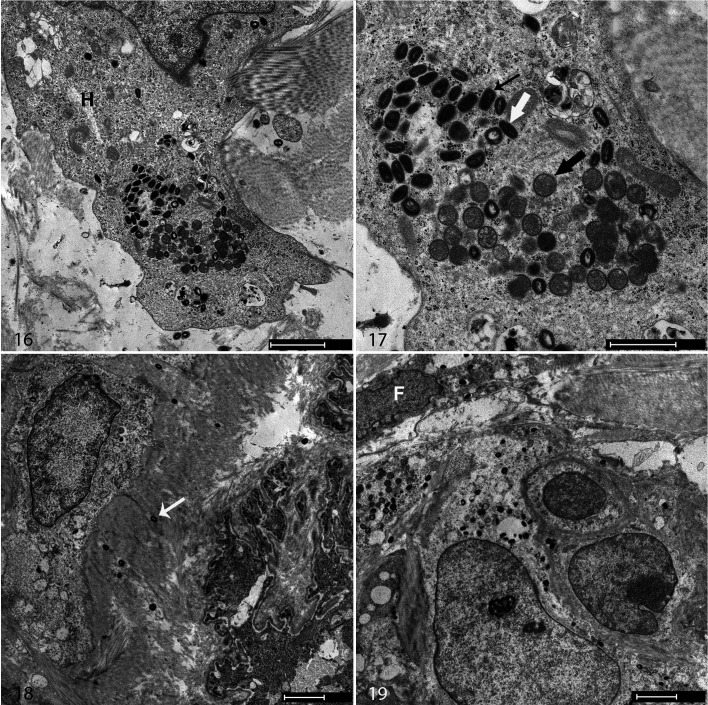
**Figures 16–19.** Lumpy skin disease, skin, calf No. 3, 11 days postinoculation. Transmission electron microscopy. Virions are present in large histiocytic-like cells (H, Fig. 16) and smaller fibroblast-like cells (F, Fig. 19). Immature particles (black thick arrow, Fig. 17), intracellular mature virions (white arrow), and brick-shaped intracellular enveloped virions (black thin arrow) are present in the cytoplasm of a large cell. Extracellular virions are also present (white arrow, Fig. 18). Scale bar in Figure 16 is 2 µm, Figure 17 is 1 µm, Figure 18 is 2 µm, and Figure 19 is 2 µm.

## Discussion

This study has characterized the gross, microscopic, and ultrastructural pathology associated with experimental lumpy skin disease. Inoculation of 4 calves with a field strain of LSDV resulted in the development of characteristic skin lesions in 2 animals. This morbidity is consistent with previous studies,^9,18^ which also found approximately 50% of challenged animals develop clinical LSD. The reasons underpinning the variable susceptibility to LSDV inoculation are unclear. This feature is peculiar to LSDV and not mirrored in experimental models of other poxviruses, including the other 2 species of capripoxvirus—sheeppox virus and goatpox virus.^4,12^

The clinical signs and gross lesions of LSD in the 2 affected calves from our study were consistent with previous descriptions of LSD. In addition, we identified targetoid lesions on the skin of the affected calves. Cutaneous targetoid lesions are characterized by a central red zone, surrounded by a pale ring of edema, and an erythematous concentric border.^23^ Targetoid lesions are associated with autoimmune diseases such as erythema multiforme, paraneoplastic syndromes, and drug reactions and often attributed to an underlying vasculitis.^13^

The key histopathological feature of LSD in our study was necrotizing vasculitis of dermal blood vessels, particularly the deep dermal plexus. Vasculitis has been described previously in studies of LSDV.^1,18,21^ Vascular changes were described by Prozesky and Barnard^18^ in experimentally infected cattle using light and ultrastructural microscopy. The changes were described as “vasculitis and lymphangitis with concomitant thrombosis and infarction resulting in oedema and necrosis.” Using EM, the authors identified virions in pericytes and endothelial cells as well as other cell types. More recent articles have described histological changes occurring in naturally infected cattle. Tageldin and colleagues^21^ examined tissues from cattle with LSD in the Sultanate of Oman. They described prominent vascular changes present in the skin lesions, including vasculitis, perivasculitis, and perivascular necrosis with concomitant thrombosis. These authors also described thickening of the tunica media and narrowing of the blood vessel lumen. Abdallah and colleagues^1^ studied lesions from cattle in Sharkia province, Egypt, and identified dermal blood vessels that were thickened and diffusely infiltrated with inflammatory cells. Vasculitis has also been described in studies of sheeppox and goatpox,^10,11^ but no other poxvirus infections are previously reported to cause vasculitis.

The cutaneous vasculitis noted in this study was associated with infarction, resulting in the well-circumscribed areas of cutaneous necrosis seen grossly. These necrotic sequestra can slough during the chronic stages of the disease, predisposing animals in the field to secondary bacterial infections and myiasis, thereby increasing mortality rates. The classic pox-like epidermal lesions of ballooning degeneration of keratinocytes, spongiosis, and intracytoplasmic inclusion bodies were seen in some early lesions; however, a number of sections contained only dermal lesions of vasculitis, inflammation, and necrosis, overlaid by a normal epidermis. This suggests the primary lesion in LSD is dermal vasculitis rather than epidermal changes. The other well-documented viral cause of vasculitis in cattle is malignant catarrhal fever caused by ovine herpesvirus 2^16^ where the lesions are lymphocytic rather than histiocyte-dominated and are found in multiple organs rather than focused almost entirely on the skin, as seen in LSDV.

Vasculitis was noted in a small number of vessels (<10) in organs other than the skin and associated musculature, including the kidney, small intestine, and cardiac muscle. There was no ischaemic change associated with these histological changes. The mild vasculitis in these organs represented the only extracutaneous lesions noted in our study. Extracutaneous LSDV lesions were inconsistent in previous descriptions, with some publications reporting mucosal lesions (erosions and ulcers) in the respiratory and gastrointestinal tracts.^14,18,21^ In contrast, extracutaneous lesions are common in experimental and field cases of sheeppox and goatpox,^6^ highlighting another distinction between the CPPV species.

Light microscopy revealed the major inflammatory cells in the dermis were large histiocytes. Immunohistochemistry using an antibody recognizing CD68 labeled approximately half of these cells, suggesting a mixed population of histiocytic cells, possibly at different stages of maturity, were present. Immunohistochemistry was also used in conjunction with a monoclonal antibody (mAb) raised against LSDV to identify the location of the virus in tissues. Labeling was occasionally seen in the cytoplasm of macrophages, keratinocytes, and epithelial cells lining hair follicles. Overall, surprisingly few cells labeled with the LSDV mAb. It is unclear if this is due to a problem with masking of the LSDV antigen in the tissues or if there is genuinely very little virus present in the lesions. Quantification of virus levels in tissue using viral titration will likely provide more information on this topic.

Electron microscopy revealed LSDV virions in a range of cell types in the dermis, clearly showing developing virions embedded in viral factories, mature particles in the cell cytoplasm, virions at the plasma membrane, and extracellular virions. Previous work has described LSDV virions in endothelial cells, pericytes, and neural tissue,^18^ but this was not a feature of the tissue we examined from our study. This difference may be due to the different virus strains used, different experimental design, or variation between animals.

This work describes the temporal pathological changes occurring in an experimental bovine model of LSD. It provides new insights into the pathogenesis of LSDV, highlights LSDV as an important differential diagnosis for cutaneous vasculitis associated with nodules in cattle, and calls attention to key differences between LSDV, sheeppox virus, and goatpox virus. Given the recent emergence of LSDV into Europe and Asia, it is important for farmers, veterinarians, and pathologists to be able to recognize this disease, particularly early lesions.

## Supplemental Material

Supplemental Material, Fig_1-5_alt - Lumpy Skin Disease Is Characterized by Severe Multifocal Dermatitis With Necrotizing Fibrinoid Vasculitis Following Experimental Infection Supplemental Material, Fig_1-5_alt for Lumpy Skin Disease Is Characterized by Severe Multifocal Dermatitis With Necrotizing Fibrinoid Vasculitis Following Experimental Infection by Beatriz Sanz-Bernardo, Ismar R. Haga, Najith Wijesiriwardana, Philippa C. Hawes, Jennifer Simpson, Linda R. Morrison, Neil MacIntyre, Emiliana Brocchi, John Atkinson, Andy Haegeman, Kris De Clercq, Karin E. Darpel and Philippa M. Beard in Veterinary Pathology

Supplemental Material, VET-19-FLM-0193_18Feb2020_marked_up - Lumpy Skin Disease Is Characterized by Severe Multifocal Dermatitis With Necrotizing Fibrinoid Vasculitis Following Experimental Infection Supplemental Material, VET-19-FLM-0193_18Feb2020_marked_up for Lumpy Skin Disease Is Characterized by Severe Multifocal Dermatitis With Necrotizing Fibrinoid Vasculitis Following Experimental Infection by Beatriz Sanz-Bernardo, Ismar R. Haga, Najith Wijesiriwardana, Philippa C. Hawes, Jennifer Simpson, Linda R. Morrison, Neil MacIntyre, Emiliana Brocchi, John Atkinson, Andy Haegeman, Kris De Clercq, Karin E. Darpel and Philippa M. Beard in Veterinary Pathology
