# Supplementary figures and images for: Lumpy Skin Disease Is Characterized by Severe Multifocal Dermatitis With Necrotizing Fibrinoid Vasculitis Following Experimental Infection
Source: Vet Pathol. 2020 Apr 21;57(3):388–96. doi: 10.1177/0300985820913268 (PMC7201124; doi:10.1177/0300985820913268)

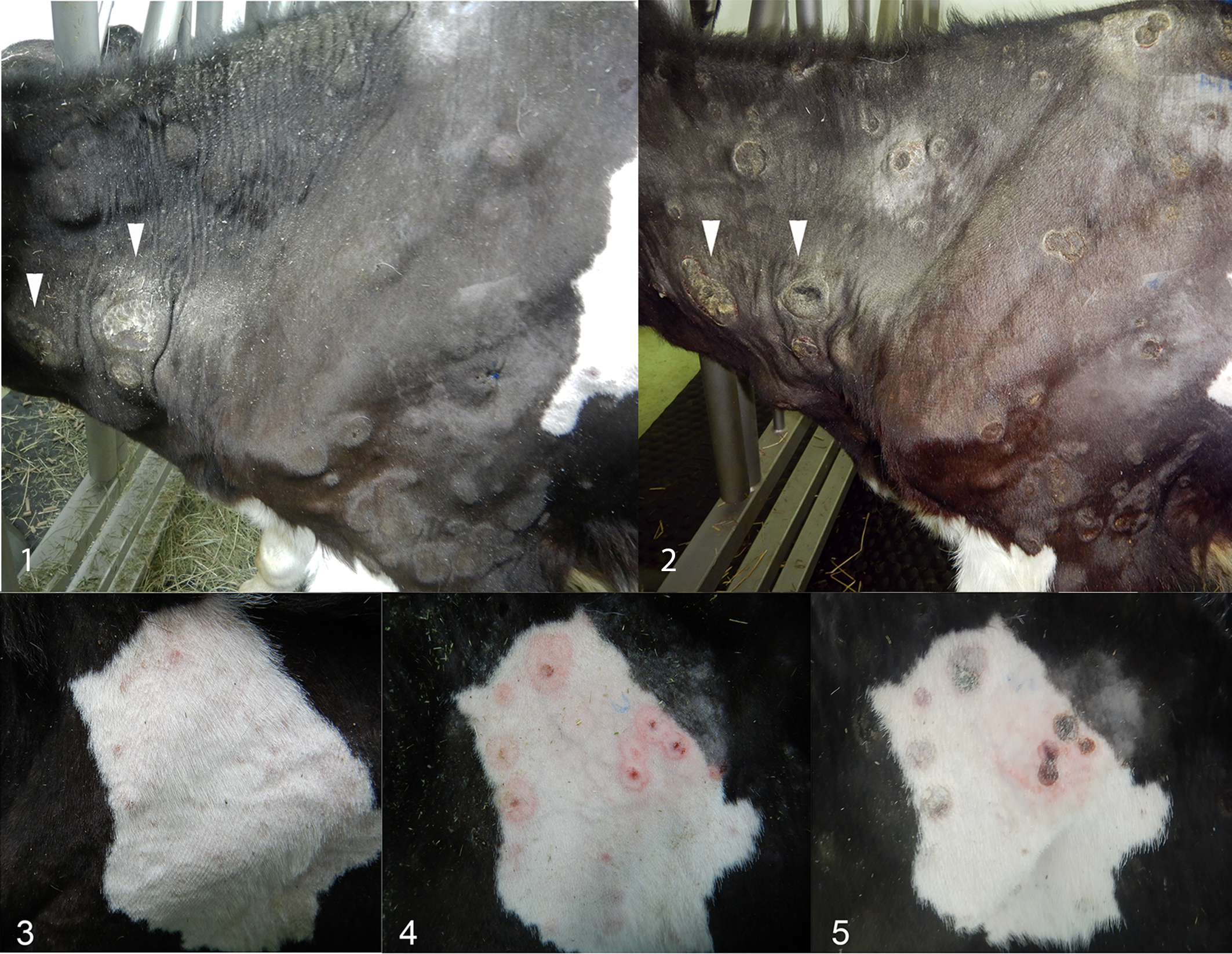

Supplement: Supplemental Material, Fig_1-5_alt - Lumpy Skin Disease Is Characterized by Severe Multifocal Dermatitis With Necrotizing Fibrinoid Vasculitis Following Experimental Infection [file Fig_1-5_alt.tif]
