# Supplementary material for: Lumpy Skin Disease Is Characterized by Severe Multifocal Dermatitis With Necrotizing Fibrinoid Vasculitis Following Experimental Infection
Source: Vet Pathol. 2020 Apr 21;57(3):388–96. doi: 10.1177/0300985820913268 (PMC7201124; doi:10.1177/0300985820913268)
Supplement: Supplemental Material, VET-19-FLM-0193_18Feb2020_marked_up - Lumpy Skin Disease Is Characterized by Severe Multifocal Dermatitis With Necrotizing Fibrinoid Vasculitis Following Experimental Infection [file VET-19-FLM-0193_18Feb2020_marked_up.docx]

Lumpy skin disease is characterised by severe multifocal dermatitis with necrotising fibrinoid vasculitis following experimental infection

Beatriz Sanz-Bernardo, Ismar R Haga, Najith Wijesiriwardana, Philippa C Hawes, Jennifer Simpson, Linda R Morrison, Neil MacIntyre, Emiliana Brocchi, John Atkinson, Andy Haegeman, Kris De Clercq, Karin E Darpel, Philippa M Beard.

Addresses:

The Pirbright Institute, Ash Rd, Surrey, GU24 0NF, UK (BSB, IRH, NW, PCH, JS, KED, PMB)

The Roslin Institute / Royal (Dick) School of Veterinary Studies, University of Edinburgh, Easter Bush, Midlothian, EH25 9RG, UK (LRM, NM, PMB)

Istituto Zooprofilattico Sperimentale della Lombardia e dell’Emilia-Romagna (IZSLER), Via Bianchi, 9, 25124 Brescia, Italy (EB)

Sciensano, Exotic and Particular Diseases, Groeselenberg 99, 1180, Ukkel, Belgium (AH, KDC)

MSD Animal Health, Walton Manor, Walton, Milton Keynes MK7 7AJ, UK (JA)

Corresponding author: PMB. The Pirbright Institute, Ash Rd, Surrey, GU24 0NF, UK 01483 232441, pip.beard@pirbright.ac.uk

**Abstract**

Lumpy skin disease is a high-consequence disease in cattle caused by infection with the poxvirus lumpy skin disease virus (LSDV). The virus is endemic in most countries in Africa and an emerging threat to cattle populations in Europe and Asia. As LSDV spreads into new regions it is important that signs of disease are recognised promptly by animal care-givers. This study describes the gross, microscopic and ultrastructural changes which occur over time in cattle experimentally challenged with LSDV. Four calves were inoculated with wildtype LSDV and monitored for 19-21 days. At 7 days after inoculation, two of the four cattle developed multifocal cutaneous nodules characteristic of LSD. Some lesions displayed a targetoid appearance. Histologically, intercellular and intracellular oedema was present in the epidermis of some nodules. Occasional intracytoplasmic inclusion bodies were identified in keratinocytes. More severe and consistent changes were present in the dermis, with marked histiocytic inflammation and necrotising fibrinoid vasculitis of dermal vessels, particularly the deep dermal plexus. Chronic lesions consisted of full-thickness necrosis of the dermis and epidermis. Lesions in other body organs were not a major feature of LSD in this study, highlighting the strong cutaneous tropism of this virus. Immunohistochemistry and electron microscopy identified LSDV-infected histiocytes and fibroblasts in the skin nodules of affected cattle. This study highlights the noteworthy lesions of LSDV and how they develop over time.

**Key words**

Bovine, dermatitis, lumpy skin disease virus, poxviridae, skin, transboundary animal diseases, vasculitis

Poxviruses are large double-stranded DNA viruses that replicate in the cytoplasm of the cell. They cause disease in a wide range of animals and humans. The most famous poxvirus is the orthopoxvirus variola virus, the causative agent of the human disease smallpox. Poxviruses of veterinary importance include orf virus in sheep and goats, myxoma virus in rabbits, fowlpox virus in poultry, and the three capripoxvirus species.

The capripoxvirus genus contains three species of poxvirus which cause high-consequence transboundary disease in ruminant livestock. Sheeppox virus and goatpox virus cause severe disease in sheep and goats, while lumpy skin disease virus (LSDV) causes disease in cattle and water buffalo. The three species are highly host-specific and of particular concern to rural communities in Africa and Asia where outbreaks of disease contribute to food insecurity.

LSDV has traditionally been found in Africa. During the 2012-2018 Eurasian LSD epidemic the virus expanded its geographical range into the Middle East and Europe, causing disease in countries including Iran, Iraq, Jordan, Turkey, Russia, Kazakhstan, Greece, Albania, Serbia and Bulgaria. The morbidity and mortality associated with this epidemic were reported at 9-26% and 0.5-2%. ^20,19,2,3,22^ In comparison, data from LSD outbreaks over 15 years in an endemic area in Uganda reported a lower disease impact with 4.77% morbidity and 0.03% mortality. ^17^

The characteristic clinical sign of LSD is distinctive and numerous raised cutaneous lesions from 0.5 to 5 cm in diameter which develop over 3-4 days from macules to papules to nodules. The cutaneous lesions are often accompanied by oral, nasal and ocular discharge, lethargy, anorexia, and in lactating animals a rapid drop in milk production. Brisket oedema and superficial lymphadenopathy are also reported. After 1-2 weeks the skin nodules become necrotic and the centre eventually sloughs. ^18,8,21,5,14,1^ The aim of this study was to describe the gross, microscopic and ultrastructural pathology occurring in cattle experimentally inoculated with LSDV.

**Materials and Methods**

Ethical statement:

This work was conducted under license P2137C5BC from the UK Home Office according to the Animals (Scientific Procedures) Act 1986. The study was approved by the Pirbright Institute Animal Welfare and Ethical Review Board.

Virus.

The LSDV strain used in this study was sourced from the OIE Capripoxvirus Reference Laboratory at Pirbright and originated from the skin of a LSD-affected bovine in eastern Europe in 2016. The virus was grown on MDBK cells (ATCC code CCL-22) in high-glucose Dulbecco’s modified Eagle’s medium (DMEM, Life Technologies # 41965) supplemented with 2.5% fetal bovine serum (FBS; Antibody Production Services Ltd, Bedford, UK), and 50 μg/ml penicillin-streptomycin (Life Technologies #15140122) at 37°C in a 5% CO_2_ atmosphere. Infectious virus titre (number of plaque-forming units/millilitre (PFU/ml)) was determined by plaque assay on MDBK cells.

Antibodies.

The monoclonal antibody targeting LSDV (2C6) was secreted into the supernatant of a hybridoma culture maintained in a CELLine disposable bioreactor (Integra). The 2C6 antibody hybridoma was generated by immunising a mouse with inactivated, partially purified LSDV (strain Neethling) then following standard protocols to generate hybridomas. Primary screening of hybridoma supernatants was by indirect ELISA against the homologous antigen and secondary screening was by immunofluorescence on LSDV-infected MDBK cells. Clone 2C6 provided strong specific labelling of LSDV-infected cells.

Animal study.

Five male castrated Holstein-Friesian calves 133-149 days old (weight range 82-112kg) were included in the study. The animals were sourced from a commercial high health herd and confirmed as negative for BVDV via PCR prior to study commencement. The animals were housed in one room (22m^2^) in a high containment (SAPO4) animal facility at The Pirbright Institute. Bedding material was provided (<https://www.mayofarmsystems.co.uk/mayo-mattress-stable-mat/>), light /dark cycle was 12:12h, temperature was held between 10-24°C, and humidity 40-70%. Animals were fed concentrated rations twice daily and given ad lib access to hay and water. Environmental enrichment was provided including rubber toys and a hollow ball stuffed with hay.

Four of the five animals (calves #2-5) were randomly assigned to the treatment group and the remaining animal (calf #1) to the untreated group. The four treated animals were each inoculated with 3ml of a LSDV suspension at a concentration of 1x10^6^ PFU/ml. 2ml (2x10^6^ PFU) was inoculated intravenously (IV) into the jugular vein, and 1ml (1x10^6^ PFU) injected intradermally (ID) into two sites on each side of the neck (0.25ml in each site). The untreated animal was not inoculated. Each animal was examined daily for clinical signs including fever, anorexia, depression, and for gross lesions including cutaneous nodules and lymphadenopathy.

Skin biopsies were carried out on the four inoculated animals at 5, 9, 11, 15, 17 and 19 days post inoculation (DPI). Hair was removed from the biopsy site with electric clippers and cleaned with skin wipes containing 2% chlorhexidine in 70% alcohol (Clinell®, GAMA Healthcare). 2.5ml of lignocaine (Lidocaine Hydrochloride injection 2%, Hameln Pharmaceuticals) was injected subcutaneously and after 10 min a 0.8cm punch biopsy taken using a disposable biopsy punch (Integra® Miltex®). One half of the biopsy tissue was placed into 10% sterile buffered formalin (Merck) for a minimum of 48 h. One quarter was placed into 4% paraformaldehyde (Santa Cruz Biotechnology, sc-281692). The remaining quarter was stored at -80^O^C for future studies. Insects were fed on the skin of the four inoculated cattle at up to 7 time points during the study. The results of this procedure are reported separately (manuscript in preparation).

The five animals were euthanized at 19-21 DPI with an overdose of barbiturate solution (Dolethal 200mg/ml Solution for injection, Vetoquinol). A post mortem examination was carried out and tissue samples collected into 10% sterile buffered formalin for a minimum of 48 h before processing.

Histopathology and Immunohistochemistry.

Tissues were processed to paraffin wax blocks, sectioned at 4 μm, and stained with hematoxylin and eosin (H&E). Martius scarlet blue trichrome stain method was taken from ^7^ and originally described in ^15^.

For immunohistochemistry to label LSDV, paraffin sections 4μm thick were cut onto Superfrost Ultra Plus microscope slides (ThermoScientific), dried overnight at 40^o^C then heated at 60^o^C for 25 min. Sections were hydrated through xylene (3 x 2 min), ethanol (3 x 2 min), and distilled water (2 min) before being rinsed in TBST (Lab Vision™ Tris Buffered Saline and Tween 20, Thermofisher TA-999-TT) 3 x 2 min. Sections were then incubated overnight with the primary antibody (LSDV monoclonal antibody 2C6 diluted 1/15 in antibody diluent (Leica code AR9352)), or a negative control of antibody diluent alone. Peroxidase-Blocking Solution (Dako REAL code S2023) was added for 10 min, the sections rinsed in TBST for 3 x 2 mins, and a secondary anti-mouse antibody conjugated to horse-radish peroxidase (Dako REAL EnVision, code K4007) added for 40 min at room temperature. The sections were again rinsed in TBST 3 x 2 mins, DAB+ Chromagen diluted in substrate buffer (20μl in 1000μl) (Dako, code K3468) was added for 10 min before a final rinse in TBST 3 x 2mins prior to counterstaining with Harris Haematoxylin (20 sec). Sections were then dehydrated, cleared and mounted.

For immunohistochemistry to label CD68, paraffin-embedded tissues section 4μm thick were prepared and hydrated through xylene (3 x 2 min), ethanol (3 x 2 min), and distilled water (2 min). Antigen retrieval was performed using proteinase K (Dako code S3020) (20 min) at room temperature, then sections were rinsed in TBST for 3 x 2 mins. The primary antibody (Dako mouse anti-CD68 clone EBM11 (code M0718)) was diluted 1/25 in TBST and incubated on the section overnight at 4^o^C. The remaining steps were as described above for the LSDV labelling.

Electron microscopy.

After 18 d storage in 4% paraformaldehyde at 4⁰C, biopsy tissues were cut into 0.5mm^3^ pieces under EM fixative (2% glutaraldehyde, Agar Scientific, in phosphate buffer) using a dissecting microscope in a fume hood. Tissue pieces were left at room temperature overnight in glutaraldehyde before further processing. The next day, following a 90 min osmium tetroxide wash (Agar Scientific), samples were thoroughly dehydrated in a graded series of ethanols (70% 45 min, 90% 15 min, 3 x 100% 15 min each) at room temperature, and the ethanol then replaced with propylene oxide (Agar Scientific). Infiltration started with 60 min wash in 50% propylene oxide/50% fresh Agar 100 epoxy resin (Agar Scientific) at room temperature with agitation. After a further 60 min in pure resin, samples were placed into Beem capsules (Agar Scientific) and polymerised at 60⁰C overnight. Thin sections (70nm) were cut using a Leica UC6 ultramicrotome, stained with uranyl acetate and lead citrate in a Leica EM Stain and imaged at 100kV in a FEI T12 TEM with Tietz 2k x 2k CCD camera.

**Results**

Clinical findings

Four calves (#2-5) were inoculated with 3 x 10^6^ PFU of wildtype LSDV via ID and IV routes, and housed with an uninoculated sentinel calf (#1) for the duration of the experiment. The four ID inoculation sites on the necks of calves #2-5 were measured each day. A firm, well-circumscribed, raised cutaneous nodule formed at each inoculation site and developed to approximately 4cm in diameter on each calf by 7 DPI. After 7 DPI the inoculation sites on calves #2 and #4 reduced in size and in some cases became inapparent. In calves #3 and #5 the inoculation sites increased up to 8cm diameter (Figure 1 and 2) and developed a well-circumscribed sunken necrotic centre which sloughed off by the third week of the experiment.

The uninoculated calf #1 did not develop any cutaneous lesions during the experiment. Calves #2 and #4 did not develop any cutaneous lesions apart from the nodules at the sites of intradermal virus inoculation on the neck. In contrast calves #3 and #5 developed numerous cutaneous nodules all over the body, particularly on the rostrum, neck, dorsal and lateral body, and legs. Nodules first appeared at 7 DPI (#3) and 6 DPI (#5) and continued to appear throughout the study until euthanasia at 19 DPI (calf #3) or 20 DPI (calf #5). The skin nodules were initially slightly raised, firm, faintly red areas that developed into raised, firm, well-circumscribed nodules up to 3cm diameter which, over 1-2 weeks, formed a dried, dark red to black sunken central necrotic region which began to peel off (Figure 1 and 2). A minority of the cutaneous lesions displayed a targetoid appearance, with a red centre less than 0.5cm diameter surrounded by a zone of lighter pink discolouration up to 1 cm in diameter, surrounded by a well demarcated dark red line (Figure 3-5). The targetoid lesions remained flat and eventually became discoloured dark brown to black, consistent with necrosis.

All four inoculated calves exhibited moderately to markedly enlarged prescapular lymph nodes which became apparent 5 DPI (calves #2 and #3) and 4 DPI (calves #4 and #5). Generalised lymphadenopathy was not a feature of the disease in this study.

Between 7 and 9 DPI all four inoculated calves exhibited a spike in temperature, rising from a baseline of approximately 39^o^C to a maximum of 40.6^o^C. Calf #1 (uninoculated control) did not display this temperature spike. The temperature of the non-clinical animals (#2 and #4) returned to baseline by 10 DPI, however the temperature of the clinically affected calves (#3 and #5) remained high for the remainder of the study. In addition to the cutaneous nodules and pyrexia, the clinically affected calves #3 and #5 exhibited intermittent mild to moderate depression and mild anorexia from 7 DPI to euthanasia, necessitating treatment with non-steroidal anti-inflammatory drugs. Calf #3 was treated on days 9, 12-14 and 18. Calf #5 was treated on days 11-13 and 19. Treatment with non-steroidal anti-inflammatory drugs did not consistently result in a reduction in temperature in the calves but did improve the general depression shown by both calves. The body condition score of both calf #3 and #5 dropped from 3/5 to 2/5 during the experiment. Both animals exhibited mild intermittent nasal discharge and mildly increased salivation. No other clinical signs were noted.

Gross lesions.

The five calves were euthanized at 19 – 21 DPI and a post mortem examination carried out. Calves #3 and #5 both had multifocal, numerous (greater than 50), well-circumscribed, flat to slightly raised, round, firm, red to purple to black, occasionally coalescing, cutaneous nodules up to 3cm diameter. The nodules were particularly numerous on the neck, dorsal and lateral body, and legs of both animals. Nodules were occasionally present in less obvious areas on calf #5, for example the skin adjacent to the anus, and within the left external ear canal.

Calf #5 had no nodules present in any other body system. Calf #3 had one nodule (1cm^3^) present within slightly oedematous subcutaneous tissues in the region of the brisket. When the musculature underlying the oedematous brisket region was incised, an additional small (<2cm^3^), focal, red and slightly firm region was found within the muscle tissue.

All five calves had mild, multifocal crusting lesions on the head and neck consistent with dermatophytosis (ringworm) which had been present and unchanged since the animals arrived , and varying degrees (mild to moderate) of cranioventral bronchopneumonia. Both were considered incidental findings.

Histopathology.

0.8cm diameter round skin biopsies (“punch” biopsies) were taken under local anaesthesia from calves #2-5 on 5, 9, 11, 15, 17, 19 and 21 DPI, or until euthanasia. The biopsies sampled cutaneous nodules on calves #3 and #5, and the corresponding area of normal skin on calves #2 and #4. The biopsies taken from calves #2 and #4 displayed no significant findings. The biopsies taken from the unaffected skin of calves #3 and #5 at 5 DPI were histologically normal. Biopsies taken from skin nodules of calves #3 and #5 from 9 DPI onwards showed lesions consistent with poxvirus infection. In the epidermis of some sections, there was ballooning degeneration indicative of intracellular edema. Keratinocytes had abundant clear to pale eosinophilic cytoplasm and shrunken hyperbasophilic nuclei, and there was microvesicle formation. Spongiosis was also present, including widening of the intercellular spaces and rupture of intercellular bridges (Figure 6). In addition, necrotic keratinocytes were present within the epidermis, scattered either singly or in small clusters, and were characterised by shrunken appearance and hypereosinophilic cytoplasm and hyperbasophilic nucleus. A minority of the degenerate and necrotic keratinocytes contained a round to spherical, pale to intensely eosinophilic, well-defined intracytoplasmic inclusion body (Figure 7 and 8). Similar changes of epithelial cell degeneration and necrosis, intracellular and intercellular oedema and intracytoplasmic inclusion bodies were occasionally noted in the epithelium lining hair follicles.

The dermis of early nodular lesions from calves #3 and #5 was infiltrated with large numbers of large round to elongated histiocytic cells with a large oval, lightly basophilic nucleus and abundant cytoplasm. The histiocytic cells were accompanied by fewer lymphocytes and plasma cells with occasional mild to moderate oedema and haemorrhage.

There was marked perivascular accumulation of large histiocytic cells and fewer lymphocytes and plasma cells throughout dermis and particularly focused on the deep cutaneous plexus at the dermal/hypodermal junction. In some sections the inflammatory cells trafficked into the blood vessel wall, and were associated with accumulation of fibrin, as identified by Martius scarlet blue trichrome stain (vasculitis) (Figure 9-11).

Necrosis was a prominent feature of subacute and chronic cutaneous nodules. In well-developed lesions the dermis and epidermis were entirely replaced by a large well-circumscribed, wedge-shaped block of necrotic tissue exhibiting widespread loss of cellular detail (necrotic sequestra). Large numbers of histiocytic cells and fibroblasts were present at the periphery of the wedge of necrotic tissue.

Samples of skin taken at post mortem examination showed similar changes to the ante mortem biopsies, with a spectrum of lesions from abundant histiocytic inflammation in the dermis and deep cutaneous plexus to regionally extensive wedge-shaped areas of epidermal and dermal necrosis (necrotic sequestra) (Figure 12).

There were subtle changes identified in other body systems. In calf #3, a nodule within the musculature of the brisket region revealed changes consistent with LSDV infection, including vasculitis, necrosis and intracytoplasmic inclusion bodies within large histiocytic cells and myocytes. In calf #5, lesions of small to medium sized blood vessels were identified in the kidney, cardiac muscle (Figure 13) and small intestine. These changes were characterised by perivascular accumulation of large histiocytic cells and fewer lymphocytes. In some cases, the inflammatory cells invaded the wall of the blood vessels and were occasionally accompanied by fibrin accumulation. Only a small number of blood vessels (fewer than four) in each section were affected.

Sections of tissue from the prescapular lymph node (which drained the inoculation site on the neck) contained medullary cords markedly expanded with large histiocytic cells. Germinal centres were prominent and contained tightly packed lymphocytes. There were a small number of necrotic areas.

In summary, histological study of tissues from calves experimentally infected with LSDV revealed a spectrum of skin lesions with changes in the epidermis and dermis and, less commonly, other tissues.

Immunohistochemistry

An antibody targeting the CD68 molecule on monocyte lineage cells was used to characterise the abundant histiocytic cells identified in the LSDV lesions. Approximately one third of the histiocytic cells in the dermis labelled strongly with the antibody to CD68+ (Figure 14). A novel anti-LSDV mAb was used to identify cells infected with the virus. Cytoplasmic labelling was identified in a minority of macrophages and epithelial cells, with the labelling often strongest in cytoplasmic vacuoles (Figure 15). Sections of skin from calf #1 (not inoculated with LSDV) and a bovine with cutaneous vasculitis not associated with LSDV were used as negative controls. Neither labelled with the anti-LSDV antibody (data not shown).

Electron microscopy.

In order to examine the ultrastructural lesions, affected skin from calf #3 at 11 DPI was processed for examination by TEM. Poxvirus virions were recognised by their characteristic hourglass-shaped core surrounded by lateral bodies and enclosed in viral membranes (Figures 16-19). Virions were present in large histiocytic-like cells and smaller elongated cells (likely fibroblasts). A full spectrum of viral morphogenesis was identified within the infected cells including spherical immature forms within viral factories, intracellular mature virions with an hourglass core, brick-like intracellular enveloped virions, and extracellular enveloped virions. No virions were identified within endothelial cells.

**Discussion**

This study has characterised the gross, microscopic and ultrastructural pathology associated with experimental lumpy skin disease. Inoculation of four calves with a field strain of LSDV resulted in the development of characteristic skin lesions in two animals. This morbidity is consistent with previous studies ^9,18^ which also found approximately 50% of challenged animals develop clinical LSD. The reasons underpinning the variable susceptibility to LSDV inoculation are unclear. This feature is peculiar to LSDV and not mirrored in experimental models of other poxviruses including the other two species of capripoxvirus – sheeppox virus and goatpox virus. ^4,12^

The clinical signs and gross lesions of LSD in the two affected calves from our study were consistent with previous descriptions of LSD. In addition, we identified targetoid lesions on the skin of the affected calves. Cutaneous targetoid lesions are characterised by a central red zone, surrounded by a pale ring of edema, and an erythematous concentric border. ^23^ Targetoid lesions are associated with autoimmune diseases such as erythema multiforme, paraneoplastic syndromes, and drug reactions, and often attributed to an underlying vasculitis. ^13^

The key histopathological feature of LSD in our study was necrotising vasculitis of dermal blood vessels, particularly the deep dermal plexus. Vasculitis has been described previously in studies of LSDV. ^18,1,21^ Vascular changes were described by Prozesky and colleagues ^18^ in experimentally infected cattle using light and ultrastructural microscopy. The changes were described as “vasculitis and lymphangitis with concomitant thrombosis and infarction resulting in oedema and necrosis”. Using EM the authors identified virions in pericytes and endothelial cells as well as other cell types. More recent papers have described histological changes occurring in naturally infected cattle. Tageldin and colleagues ^21^ examined tissues from cattle with LSD in the Sultanate of Oman. They described prominent vascular changes present in the skin lesions, including vasculitis, perivasculitis and perivascular necrosis with concomitant thrombosis. These authors also described thickening of the tunica media and narrowing of the blood vessel lumen. Abdallah and colleagues studied lesions from cattle in Sharkia province, Egypt ^1^ and identified dermal blood vessels that were thickened and diffusely infiltrated with inflammatory cells. Vasculitis has also been described in studies of sheeppox and goatpox ^10,11^ but no other poxvirus infections are previously reported to cause vasculitis.

The cutaneous vasculitis noted in this study was associated with infarction, resulting in the well-circumscribed areas of cutaneous necrosis seen grossly. These necrotic sequestra can slough during the chronic stages of the disease, predisposing animals in the field to secondary bacterial infections and myiasis, thereby increasing mortality rates. The classic pox-like epidermal lesions of ballooning degeneration of keratinocytes, spongiosis, and intracytoplasmic inclusion bodies were seen in some early lesions, however a number of sections contained only dermal lesions of vasculitis, inflammation and necrosis, overlaid by a normal epidermis. This suggests the primary lesion in LSD is dermal vasculitis rather than epidermal changes. The other well-documented viral cause of vasculitis in cattle is malignant catarrhal fever caused by ovine herpesvirus 2 ^16^ where the lesions are lymphocytic rather than histiocyte-dominated, and are found in multiple organs rather than focused almost entirely on the skin as seen in LSDV.

Vasculitis was noted in a small number of vessels (less than 10) in organs other than the skin and associated musculature, including the kidney, small intestine and cardiac muscle. There was no ischaemic change associated with these histological changes. The mild vasculitis in these organs represented the only extracutaneous lesions noted in our study. Extra-cutaneous LSDV lesions were inconsistent in previous descriptions, with some publications reporting mucosal lesions (erosions and ulcers) in the respiratory and gastrointestinal tracts. ^18,14,21^ In contrast, extra-cutaneous lesions are common in experimental and field cases of sheeppox and goatpox, ^6^ highlighting another distinction between the CPPV species.

Light microscopy revealed the major inflammatory cells in the dermis were large histiocytes. Immunohistochemistry using an antibody recognising CD68 labelled approximately half of these cells, suggesting a mixed population of histiocytic cells, possibly at different stages of maturity, were present. Immunohistochemistry was also used in conjunction with a monoclonal antibody (mAb) raised against LSDV to identify the location of the virus in tissues. Labelling was occasionally seen in the cytoplasm of macrophages, keratinocytes, and epithelial cells lining hair follicles. Overall surprisingly few cells labelled with the LSDV mAb. It is unclear if this is due to a problem with masking of the LSDV antigen in the tissues, or if there is genuinely very little virus present in the lesions. Quantification of virus levels in tissue using viral titration will likely provide more information on this topic.

Electron microscopy revealed LSDV virions in a range of cell types in the dermis, clearly showing developing virions embedded in viral factories, mature particles in the cell cytoplasm, virions at the plasma membrane and extracellular virions. Previous work has described LSDV virions in endothelial cells, pericytes and neural tissue, ^18^ but this was not a feature of the tissue we examined from our study. This difference may be due to the different virus strains used, different experimental design, or variation between animals.

This work describes the temporal pathological changes occurring in an experimental bovine model of LSD. It provides new insights into the pathogenesis of LSDV, highlights LSDV as an important differential diagnosis for cutaneous vasculitis associated with nodules in cattle and calls attention to key differences between LSDV, sheeppox virus and goatpox virus. Given the recent emergence of LSDV into Europe and Asia, it is important for farmers, veterinarians and pathologists to be able to recognise this disease, particularly early lesions.

Acknowledgements

The authors thank the Pirbright Animal Services Unit for assistance with the animal experiments.

Declaration of conflicting interests

None

Funding

This work was supported by MSD Animal Health and the Biotechnology and Biological Sciences Research Council (BBSRC) grant BB/R002606/1, and strategic funding from the BBSRC to the Pirbright Institute (BBS/E/I/00007037, BBS/E/I/00007033, BBS/E/I/00007036, BBS/E/I/00002087) and the Roslin Institute (BBS/E/D/20002173).

Figure Legends.

Figure 1 and 2. Lumpy skin disease, calf #5, 12 days post inoculation (DPI) (Figure 1), and 20 DPI (Figure 2). Multiple cutaneous nodules are present. One nodule in Figure 1 was labelled with blue suture, and one nodule in Figure 2 has blue ink on the surface.Inoculation sites are indicated with arrows.

Figure 3-5. Figures 3-5 Lumpy skin disease, calf #3, right flank. Targetoid lesions are present at 7 (figure 3), 13 (figure 4) and 19 (figure 5) DPI. Blue surface labelling (ink) is present on the skin in Figure 4 and 5.

Figure 6-8: Acute lumpy skin disease, skin, calf, 9 DPI (Figure 6) and 15 DPI (Figure 7 and 8). Degeneration and necrosis of keratinocytes, intracytoplasmic inclusion bodies, and vesicles are present in the epidermis. Oedema, haemorrhage, and influx of lymphocytes and macrophages are present in the dermis. Hematoxylin and eosin.

Figure 9-11: Lumpy skin disease, skin, calf. There is a well-demarcated wedge-shaped region of necrosis (infarct) (Figure 9). In the centre of the infarct, the wall of a large muscular blood vessel is disrupted by mononuclear inflammatory cells and fibrin (fibrinonecrotic vasculitis) Figure 10, and red staining in Figure 11. Hematoxylin and eosin (Figure 9 and 10). Martius scarlet blue trichrome stain (Figure 11).

Figures 12-14. Lumpy skin disease (LSDV), calf. Figure 12. Skin. A cutaneous infarct is present, encompassing both the epidermis and dermis. Hematoxylin and eosin (HE). Figure 13. Myocardium. A medium sized blood vessel in the myocardium is surrounded by histiocytic cells and lymphocytes, with some of these mononuclear cells invading the blood vessel wall. HE. Figure 14-15: Lumpy skin disease, skin, calf. The dermis contains histiocytic cells that are immunolabelled for CD68 (Fig 14) and for LSDV (Fig. 15)

Figure 16-19: Lumpy skin disease, skin, calf #3, 11 days post inoculation (DPI). Transmission electron microscopy. Virions are present in large histiocytic-like cells (H, Fig 16) and smaller fibroblast-like cells (F, Fig 19). Immature particles (black thick arrow, Fig 17), intracellular mature virions (white arrow), and brick-shaped intracellular enveloped virions (black thin arrow) are present in the cytoplasm of a large cell. Extracellular virions are also present (white arrow, Fig 18). Scale bar in image 16 is 2µm, image 17 is 1 µm, image 18 is 2 µm and image 19 is 2 µm.

References

1. Abdallah FM, El Damaty HM, Kotb GF. Sporadic cases of lumpy skin disease among cattle in Sharkia province, Egypt: Genetic characterization of lumpy skin disease virus isolates and pathological findings. *Veterinary world*. 2018;11**:** 1150-1158.

2. Abutarbush SM, Ababneh MM, Al Zoubi IG, et al. Lumpy Skin Disease in Jordan: Disease Emergence, Clinical Signs, Complications and Preliminary-associated Economic Losses. *Transboundary and emerging diseases*. 2015;62**:** 549-554.

3. Al-Salihi KA, Hassan IQ. Lumpy Skin Disease in Iraq: Study of the Disease Emergence. *Transboundary and emerging diseases*. 2015;62**:** 457-462.

4. Babiuk S, Bowden TR, Parkyn G, et al. Yemen and Vietnam capripoxviruses demonstrate a distinct host preference for goats compared with sheep. *The Journal of general virology*. 2009;90**:** 105-114.

5. Babiuk S, Bowden TR, Parkyn G, et al. Quantification of lumpy skin disease virus following experimental infection in cattle. *Transboundary and emerging diseases*. 2008;55**:** 299-307.

6. Bowden TR, Babiuk SL, Parkyn GR, Copps JS, Boyle DB. Capripoxvirus tissue tropism and shedding: A quantitative study in experimentally infected sheep and goats. *Virology*. 2008;371**:** 380-393.

7. Carleton; HM, Drury; RAB, Wallington EA. *Carleton's Histological technique* 4ed. New York: Oxford University Press; 1967.

8. Carn VM, Kitching RP. The clinical response of cattle experimentally infected with lumpy skin disease (Neethling) virus. *Archives of virology*. 1995;140**:** 503-513.

9. Carn VM, Kitching RP. An investigation of possible routes of transmission of lumpy skin disease virus (Neethling). *Epidemiology and infection*. 1995;114**:** 219-226.

10. Embury-Hyatt C, Babiuk S, Manning L, et al. Pathology and viral antigen distribution following experimental infection of sheep and goats with capripoxvirus. *J Comp Pathol*. 2012;146**:** 106-115.

11. Gulbahar MY, Davis WC, Yuksel H, Cabalar M. Immunohistochemical evaluation of inflammatory infiltrate in the skin and lung of lambs naturally infected with sheeppox virus. *Vet Pathol*. 2006;43**:** 67-75.

12. Hajjou S, Khataby K, Amghar S, et al. Assessment and comparison of the pathogenicity of Sheeppox Virus strains isolated in Morocco. *Iran J Microbiol*. 2017;9**:** 372-380.

13. Hughey LC. Approach to the hospitalized patient with targetoid lesions. *Dermatol Ther*. 2011;24**:** 196-206.

14. Kasem S, Saleh M, Qasim I, et al. Outbreak investigation and molecular diagnosis of Lumpy skin disease among livestock in Saudi Arabia 2016. *Transboundary and emerging diseases*. 2018;65**:** e494-e500.

15. Lendrum AC, Fraser DS, Slidders W, Henderson R. Studies on the character and staining of fibrin. *Journal of clinical pathology*. 1962;15**:** 401-413.

16. O'Toole D, Li H. The pathology of malignant catarrhal fever, with an emphasis on ovine herpesvirus 2. *Vet Pathol*. 2014;51**:** 437-452.

17. Ochwo S, VanderWaal K, Munsey A, et al. Spatial and temporal distribution of lumpy skin disease outbreaks in Uganda (2002-2016). *BMC veterinary research*. 2018;14**:** 174.

18. Prozesky L, Barnard BJ. A study of the pathology of lumpy skin disease in cattle. *The Onderstepoort journal of veterinary research*. 1982;49**:** 167-175.

19. Sameea Yousefi P, Mardani K, Dalir-Naghadeh B, Jalilzadeh-Amin G. Epidemiological Study of Lumpy Skin Disease Outbreaks in North-western Iran. *Transboundary and emerging diseases*. 2016.

20. Sevik M, Dogan M. Epidemiological and Molecular Studies on Lumpy Skin Disease Outbreaks in Turkey during 2014-2015. *Transboundary and emerging diseases*. 2016.

21. Tageldin MH, Wallace DB, Gerdes GH, et al. Lumpy skin disease of cattle: an emerging problem in the Sultanate of Oman. *Tropical animal health and production*. 2014;46**:** 241-246.

22. Tasioudi KE, Antoniou SE, Iliadou P, et al. Emergence of Lumpy Skin Disease in Greece, 2015. *Transboundary and emerging diseases*. 2016;63**:** 260-265.

23. Wolf R, Lipozencic J. Shape and configuration of skin lesions: targetoid lesions. *Clin Dermatol*. 2011;29**:** 504-508.
